# PDGFRα^+^ fibroblast ablation exacerbates pathologic features in a model of house dust mite-induced allergenic asthma

**DOI:** 10.1242/dmm.052323

**Published:** 2025-12-29

**Authors:** Ha Eun Shin, Sanyeowool An, Jack Heckl, Cady Komori, Hillary Sullivan, Rodson Zorilla, Hyungdong Yoon, Te-Kie Pedro, Michelle D. Tallquist, Juwon Park

**Affiliations:** ^1^ Department of Tropical Medicine, Medical Microbiology, and Pharmacology, John A. Burns School of Medicine, University of Hawai'i at Mānoa, Honolulu, HI 96813, USA; ^2^ Cancer Research Institute, Seoul National University College of Medicine, Seoul 03080, South Korea; ^3^ Department of Cell and Molecular Biology, John A. Burns School of Medicine, University of Hawai'i at Mānoa, Honolulu, HI 96813, USA; ^4^ Program in Cancer Biology, Vanderbilt University, Nashville, TN 37232, USA; ^5^ Division of Hematology/Oncology, Department of Medicine, Vanderbilt University Medical Center, Nashville, TN 37232, USA; ^6^ Center for Cardiovascular Research, John A. Burns School of Medicine, University of Hawai'i at Mānoa, Honolulu, HI 96813, USA

**Keywords:** Asthma, House dust mite, PDGFRα^+^ fibroblast, Cell ablation, Neutrophil, Inducible bronchus-associated lymphoid tissue

## Abstract

Asthma, a chronic inflammatory airway disease, remains a major global health concern. Fibroblasts, the cell type responsible for tissue repair and fibrosis, are therefore a potential therapeutic target for asthma-related lung disease. However, the role of fibroblasts in the onset and progression of asthma is poorly understood. Thus, we sought to determine the effects of fibroblast loss on lung homeostasis and asthma development using a transgenic mouse model to ablate PDGFRα^+^ fibroblasts. We observed a consistent reduction in PDGFRα^+^ cells (75-85% in the mesenchyme), which persisted for several months. The PDGFRα^+^ fibroblast-ablated lungs exhibited a reduced number of lipofibroblasts, altered extracellular matrix gene expression and increased neutrophils in both the bronchoalveolar lavage fluid and the lung tissues under steady-state conditions. When asthma was induced, we found that loss of PDGFRα^+^ fibroblasts resulted in increased mucous production, neutrophil activation and proinflammatory cells, such as interstitial macrophages and eosinophils, which can worsen asthma. These findings highlight the essential roles of PDGFRα^+^ fibroblasts in maintaining immune balance and how their loss leads to dysregulated airway immune composition and remodeling, contributing to asthma pathogenesis.

## Teaser

**Summary:** This study demonstrates that PDGFRα^+^ fibroblast ablation worsens asthma pathology by elevating neutrophil numbers and activation, highlighting fibroblast–immune crosstalk in asthma pathogenesis.

## INTRODUCTION

Asthma is a chronic inflammatory disease characterized by airway hyper-responsiveness and airway remodeling, with involvement of epithelial cells, fibroblasts and smooth muscle cells (Boser et al., 2017; Mostaco-Guidolin et al., 2019). It affects over 262 million people worldwide and is the second leading cause of death among chronic respiratory diseases, resulting in 455,000 deaths in 2019 (GBD 2019 Diseases and Injuries Collaborators, 2020; Wang et al., 2023). Despite efforts to reduce disease burden, the overall global prevalence of asthma is estimated to increase. At present, there is no cure for asthma, and standard treatment options – including beta-agonists, corticosteroids and anti-inflammatory medications – are mainly focused on symptom management (Holgate et al., 2015; Schoettler and Strek, 2020). Although current therapies efficiently manage individuals with mild asthma, ∼8% of mild asthmatics progress to a more severe and often treatment-resistant (Szefler et al., 2002) phenotype (Chen et al., 2018). Therefore, a better understanding of the underlying disease processes is needed.

Pathological lung fibrosis is characterized by the expansion of activated fibroblasts, excessive extracellular matrix (ECM) deposition and airway remodeling (Upagupta et al., 2018; Wilson and Wynn, 2009). Fibroblasts are primarily responsible for ECM synthesis and maintenance during tissue injury and acute inflammation – processes that are beneficial to the lung homeostasis (Plikus et al., 2021; Talbott et al., 2022), but fibroblast expansion and chronic activation can lead to fibrosis, ultimately causing organ dysfunction (Humeres and Frangogiannis, 2019; Musale et al., 2023; Selman and Pardo, 2021). Although the role of fibroblasts in chronic lung fibrosis is relatively well characterized, their contributions to asthma development are not fully understood. Therefore, it is crucial to elucidate the role of fibroblasts using *in vivo* asthma models.

Platelet-derived growth factor receptor alpha (PDGFRα) is expressed in fibroblasts throughout the body (Collins et al., 2011; Endale et al., 2017; Ivey et al., 2019; Karlsson et al., 2000), and PDGFRα^+^ cells are considered the predominant, resident fibroblast population in the lung (Endale et al., 2017; Li et al., 2018; Ntokou et al., 2015; Ushakumary et al., 2021). PDGFRα^+^ cells overlap extensively with type I collagen (Col1a1)-expressing cells, providing further support that they are responsible for lung ECM production (Green et al., 2016; Hung et al., 2013). Under pathological conditions, the cellular sources of activated lung fibroblasts are diverse, including PDGFRα^+^ fibroblasts, lipofibroblasts and pericytes (El Agha et al., 2017; Hung et al., 2013; Li et al., 2018). The conversion of resting PDGFRα^+^ fibroblasts (Rock et al., 2011) to being activated has been observed with bleomycin-induced fibrosis (Rock et al., 2011) and idiopathic pulmonary fibrosis (Biasin et al., 2020). In the context of asthma, the specific involvement and contributions of PDGFRα^+^ fibroblasts have not been thoroughly explored. Given that PDGFRα^+^ fibroblasts are necessary for lung repair and remodeling, we utilized a mouse strain in which PDGFRα^+^ fibroblasts could be eliminated, and we investigated the consequences of their loss on lung homeostasis and asthma pathogenesis.

## RESULTS

### Genetic ablation of PDGFRα^+^ fibroblasts in adult lung

**Fig. 1.**
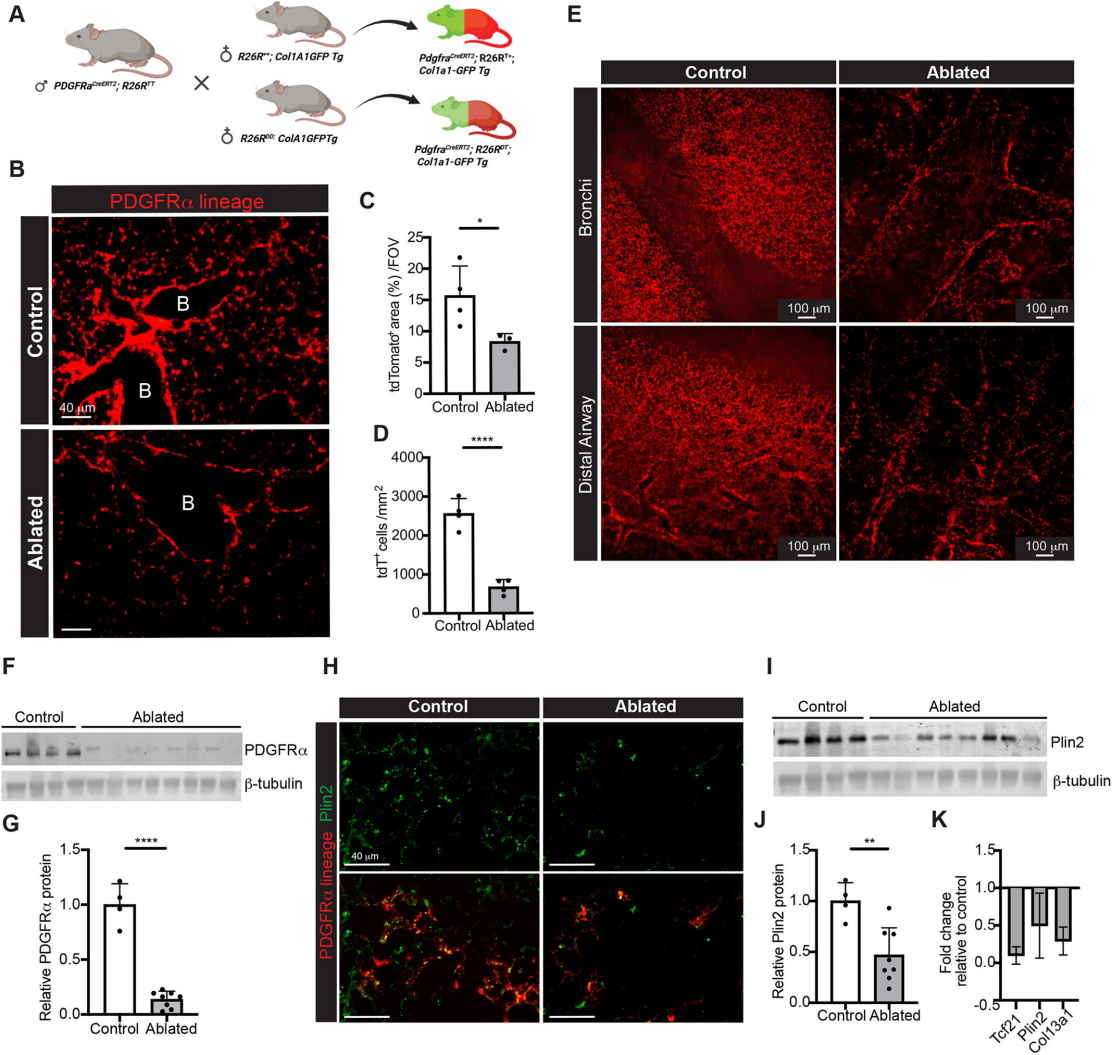
**Efficacy of PDGFRα^+^ fibroblast ablation.** (A) Breeding scheme for control and PDGFRα^+^ fibroblast-ablated (referred to as ‘ablated’) mice with fluorescence markers. Note that not all animals possessed the *Col1a1-GFP* transgene. (B) Representative images of PDGFRα lineage cells (tdTomato) in lung sections of control and ablated mice (*n*=4-5 per group). Scale bars: 40 µm. (C,D) Quantification of tdTomato lineage-traced fibroblasts: area (C) and numbers (D) of tdTomato^+^ cells in the sections (*n*=4 of each genotype with five fields per lung section). For tdTomato^+^ cells (D), images of the interstitium were used. (E) Whole-mount cleared images of the bronchial and distal airway regions in control and ablated mice. Scale bars: 100 µm. (F) Western blot for PDGFRα protein in control (*n*=4) and ablated (*n*=8) mice. (G) Densitometry of the western blot of PDGFRα protein normalized to β-tubulin. (H) Representative images of PDGFRα lineage (red) and Plin2 (green) fluorescence in control and ablated mice. Scale bars: 40 mm. (I) Western blot for Plin2 (control, *n*=4; ablated, *n*=8). PDGFRα (F) and Plin2 (I) were run on the same gel with a single shared control lane, which is shown in both panels. (J) Relative Plin2 protein expression levels, normalized to β-tubulin expression. (K) Results from quantitative PCR (qPCR) analysis of lipofibroblast transcripts, normalized to *Gapdh*. *n*=6-8. Mean±s.d. (C,D,G,J,K) Unpaired, two-tailed *t*-test. *P≤0.05; **P≤0.01; ****P≤0001.

Although PDGFRα^+^ cells constitute a majority of the lung's resident fibroblasts, it is unclear whether these cells are needed for ECM maintenance in the adult lung. Therefore, we examined the functional consequences of removing these cells. Using mice that express diphtheria toxin A (DTA) in PDGFRα-expressing cells after Cre recombination, *Pdgfra^CreERT2/+^; Gt(ROSA)26Sor^tdT/DTA^* (hereafter referred to as ‘ablated’), we were able to deplete PDGFRα^+^ cells (Chung et al., 2018). *Pdgfra^CreERT/+^* lungs were analyzed 2-7 months post-induction. To monitor *Col1a1* promoter activity, *Pdgfra^CreERT/+^* mice were also crossed with either *R26R^++^;Col1a1-GFP* or *R26R^dd^ Col1a1-GFP* mice (Fig. 1A). As expected (Hung et al., 2013; Li et al., 2018), PDGFRα^+^ fibroblasts were abundant in control, lineage-tagged adult lungs. PDGFRα lineage cells (tdTomato^+^) were observed in the interstitium, peribronchial and perivascular regions (Fig. 1B). By comparison, ablated lungs demonstrated a 40-50% reduction in tdTomato-labeled cells in the lung. Airway fibroblasts persisted but at reduced numbers (Fig. 1C,D). Three-dimensional imaging of whole-mount cleared lungs confirmed that there was significant loss of PDGFRα lineage cells. Interestingly, lineage-labeled cells were still observed along the airway (Fig. 1E). Furthermore, western blot analysis demonstrated 85% reduction in PDGFRα protein expression in the ablated lungs compared to that in the control lungs (Fig. 1F,G). These data indicate that the reduction in PDGFRα^+^ fibroblasts following the tamoxifen induction was maintained over 7 months.

### PDGFRα^+^ fibroblast ablation results in the reduction of lipofibroblasts

We and others have observed that a subset of PDGFRα^+^ cells (∼50%) are lipid-laden lipofibroblasts (Ntokou et al., 2015; Park et al., 2019). Thus, to determine whether PDGFRα^+^ fibroblast ablation impacted lipofibroblast numbers, we compared the number of perilipin-2 (Plin2)-expressing cells in the control and ablated lungs. As expected, Plin2^+^ cells and Plin2 expression were drastically reduced in the ablated lungs compared to control lungs (Fig. 1H-J). To further determine whether there was lipofibroblast reduction, the expression of *Tcf21* and collagen 13a1 (*Col13a1*), putative lipofibroblast markers (Park et al., 2019), was examined by quantitative PCR (qPCR) analysis. We found that *Tcf21*, *Plin2* and *Col13a1* gene expression was reduced in the ablated lungs compared to that in control lungs (Fig. 1K). Taken together, these data support that some lipofibroblasts are present in the adult PDGFRα^+^ fibroblast population.

### Collagen production and expression of ECM genes in baseline lung after PDGFRα^+^ cell ablation

**Fig. 2.**
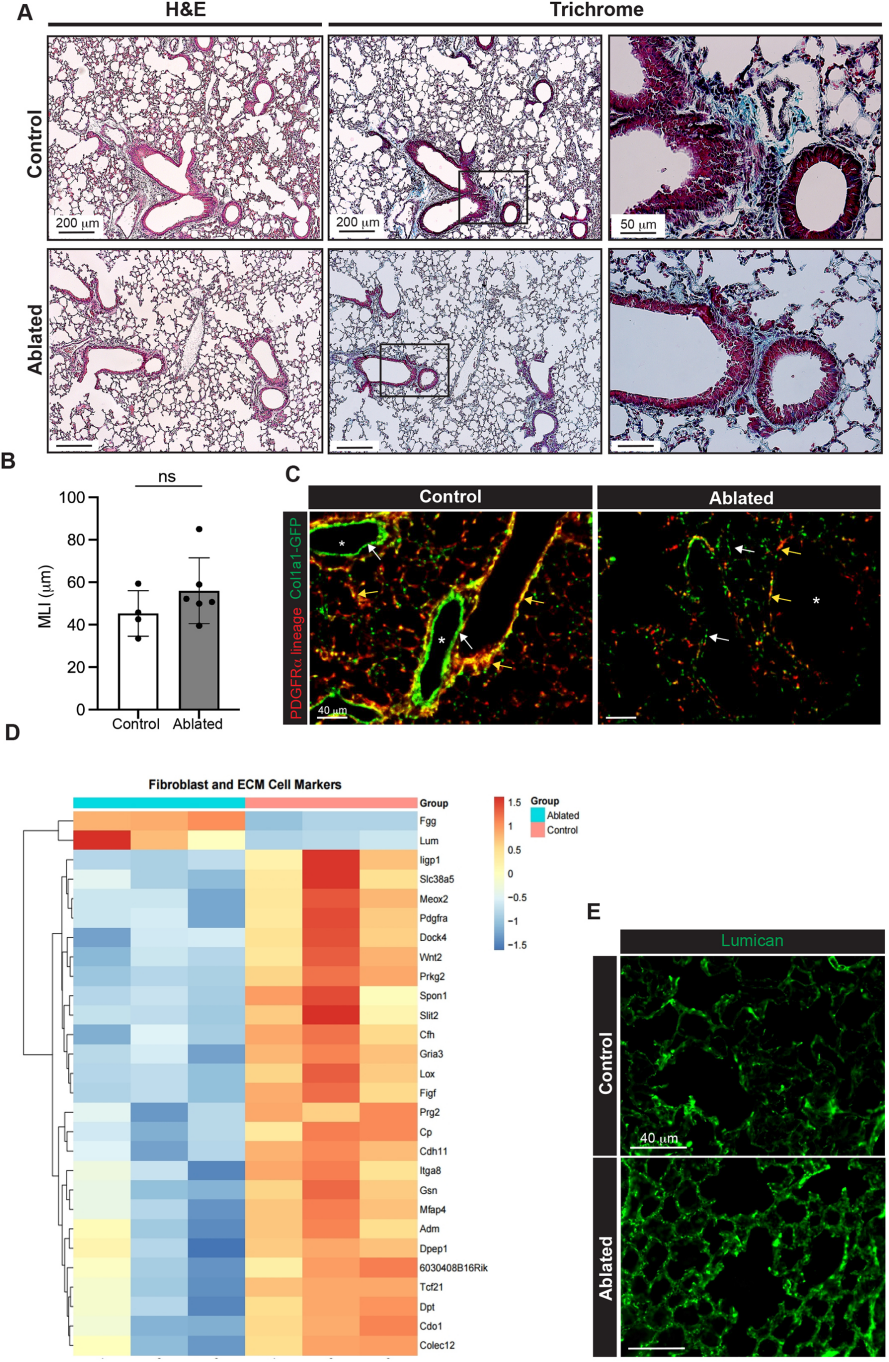
**Extracellular matrix (ECM) analyses in PDGFRα^+^ fibroblast-ablated lungs.** (A) Representative images of Hematoxylin and Eosin (H&E) (left panels) and Gomori's trichrome staining (right panels) after PDGFRα^+^ fibroblast ablation (*n*=4-5 mice per group). The right column shows magnified images of the boxed areas in the middle column, containing the airway. Scale bars: 200 µm (left and middle columns), 50 µm (right column). (B) Quantitative analyses of the airway space sizes, as measured by mean linear intercept (MLI) (*n*=4-6). (C) Representative images showing PDGFRα lineage (red) and Col1a1 reporter (green) fluorescence in lungs from control and ablated mice. Yellow arrows indicate double-positive cells, tdTomato^+^Col1a1^+^; white arrows indicate Col1a1^+^ cells. Asterisks indicate pulmonary vessels. Scale bars: 40 µm. (D) Heatmap displaying the expression of fibroblast- and ECM-related genes among differentially expressed genes (DEGs). Each column represents one sample, and each cell represents a log2-transformed robust multi-array averaging (RMA)-normalized expression value. (E) Representative immunofluorescent staining of lumican in lungs from control and ablated mice (*n*=5 per group). Scale bars: 40 µm. Mean±s.d. (B) Welch's test. ns, not significant.

We have reported that adult mice that have undergone long-term PDGFRα^+^ fibroblast ablation exhibit equivalent survival and maintain a normal lifespan compared to littermate controls, although they have been observed to grow thinner and have overall reduced body weight (Kuwabara et al., 2022). Hematoxylin and Eosin (H&E) staining showed a thinned mesenchyme and a trend toward increased mean linear intercept (MLI) in the lungs of ablated mice compared to those of control mice (Fig. 2A,B). However, there were no significant changes in total collagen content around the airways and the blood vessels (Fig. 2A). Although staining for deposited collagen revealed no significant differences between control and ablated lungs, the collagen reporter gene, *Col1a1-GFPTg*, demonstrated a drastic reduction in the number of cells with an active collagen promoter (Fig. 2C). This indicates that PDGFRα lineage fibroblasts are the key cell population responsible for collagen production in the mouse lung.

To gain further insights into transcriptional changes in the lung following PDGFRα^+^ fibroblast ablation, we performed microarray analysis. A total of 100 genes were differentially expressed; two were upregulated and 98 were downregulated in the ablated lungs compared to those in control lungs (Fig. S1). Among the differentially expressed genes (DEGs), ECM-related and fibroblast-specific genes were reduced in ablated lungs compared to those in control lungs, including *Tcf21*, *Wnt2*, *Gsn*, *Meox2*, *Mfap4* and *Pdgfra* (Burgstaller et al., 2017; Zhang et al., 2019) (Fig. 2D). We also confirmed *Col1a1* expression from the microarray data; although *Col1a1* expression tended to be reduced in the ablated lungs compared to that in control lungs, the change did not meet the thresholds of |log2 fold change (FC)|>1 and adjusted *P*<0.05. Although a majority of the DEGs were reduced in ablated lungs compared to controls, two genes, lumican (*Lum*) and fibrinogen gamma chain (*Fgg*), were increased. The increase in *Lum* transcript resulted in a similar increase in protein (Fig. 2E). Collectively, these data suggest that other fibroblast subtypes do not replace the loss of PDGFRα^+^ fibroblasts.

### Effects of PDGFRα^+^ fibroblast ablation on lung immune homeostasis

**Fig. 3.**
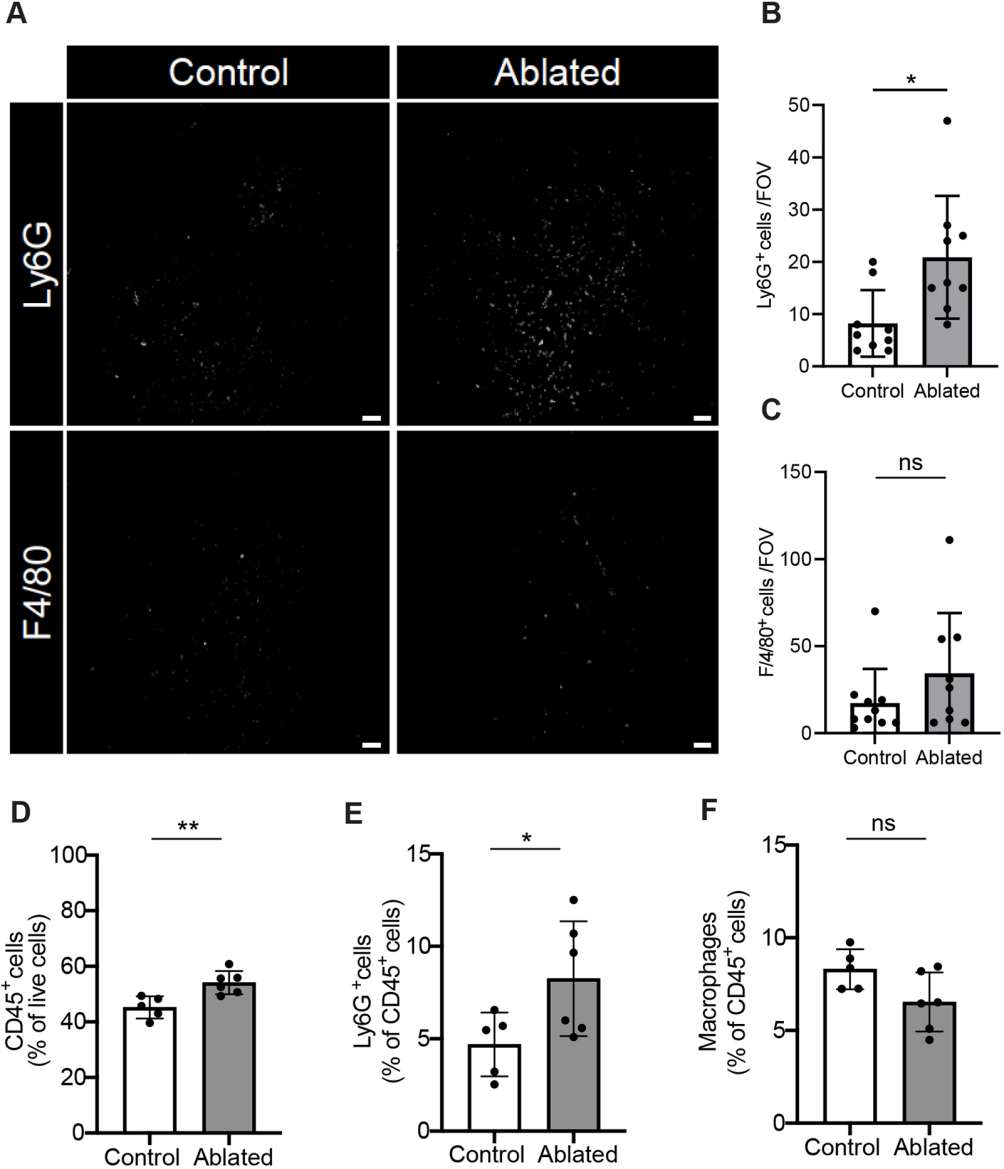
**Increased neutrophil numbers in PDGFRα^+^ fibroblast-ablated lungs.** (A) Representative immunofluorescent images of Ly6G and F4/80 expression in control and ablated lungs. Scale bars: 100 μm. (B,C) Quantification of Ly6G^+^ cells (neutrophils; B) and F4/80^+^ cells (macrophages; C) in the adult lung sections (*n*=5 mice per group, two fields per mouse, and each data point is one field). FOV, field of view. (D-F) Flow cytometry analyses of CD45^+^ cells (leukocytes; D), neutrophils (E) and macrophages (F). Mean±s.d. (B-F) Unpaired, two-tailed *t*-test. ns, not significant; *P≤0.05; **P≤0.01.

Although fibroblasts play a critical role in chronic inflammation (Plikus et al., 2021), their contribution to immune homeostasis in the steady-state lung remains relatively unexplored. Therefore, we determined the effects of PDGFRα^+^ fibroblast loss on the lung innate immune system. First, we analyzed the composition of neutrophils (Ly6G^+^ cells) and macrophages [F4/80^+^ (also known as Adgre1^+^) cells] in the lungs following PDGFRα^+^ fibroblast ablation. Ablated lungs showed elevated neutrophils, but comparable macrophage numbers, compared to the control lungs (Fig. 3A-C). Furthermore, flow cytometry analysis of lung tissues revealed that the ablated lungs had a significant increase in total leukocytes [CD45^+^ (also known as Ptprc^+^) cells] compared to the control lungs (Fig. 3D). Consistent with Ly6G and F4/80 immunofluorescence results, the ablated lungs showed a statistically significant increase in neutrophils, while macrophage levels remained comparable to those in the control lungs (Fig. 3E,F). Flow cytometric analysis of bronchoalveolar lavage fluid (BALF) showed a selective increase in neutrophils in the ablated mice, with no changes observed in eosinophils or macrophages (Fig. S2). These findings suggest that PDGFRα^+^ fibroblast ablation leads to increased neutrophil numbers in the lung.

### Effects of PDGFRα^+^ cell ablation on house dust mite-exposed lungs

**Fig. 4.**
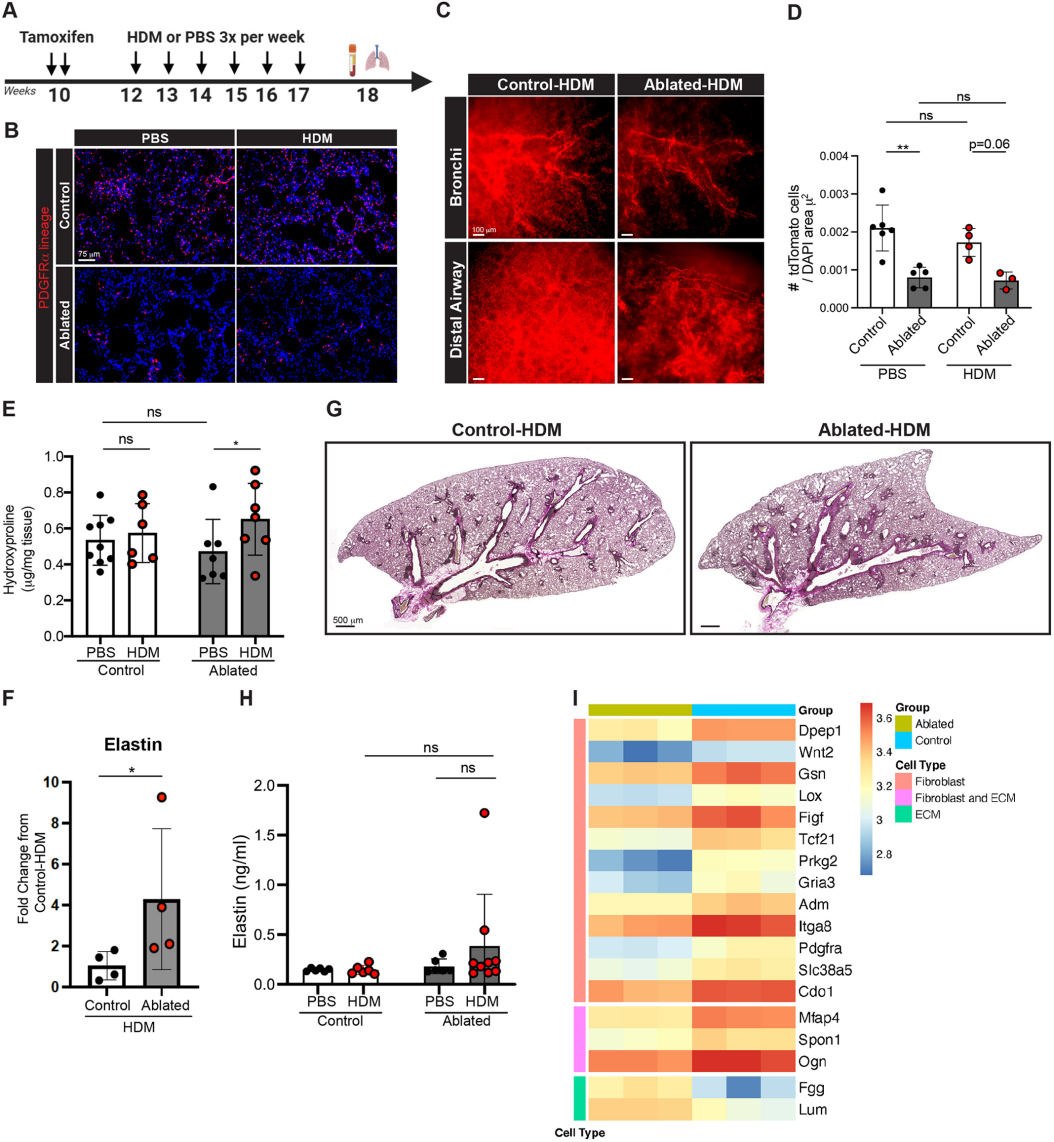
**Comparison of house dust mite (HDM)-exposed control and ablated mice.** (A) Experimental design for tamoxifen induction and HDM exposure. Control and ablated mice were exposed to either vehicle or HDM intranasally, three times per week for 5 weeks. (B) Representative images of lung sections from mice exposed to HDM or PBS. (C) Whole-mount cleared images of bronchial and distal airway regions from HDM-exposed control and ablated lung. Scale bars: 100 µm. (D) Quantification of tdTomato^+^ cells normalized to DAPI area (*n*=3-6 biological replicates). (E) Hydroxyproline content in HDM- or PBS- exposed control and ablated lungs (*n*=6-7 each). (F) qPCR analysis of elastin normalized to *Gadph*. Relative fold change compared to HDM-exposed control lungs (*n*=4-5 per group). (G) Representative images of Verhoeff Van Gieson-stained HDM-exposed control and ablated lung sections (*n*=4 per group). Scale bars: 500 µm. (H) Enzyme-linked immunosorbent assay of elastin in lung lysates. (I) Heatmap of DEGs for fibroblast and ECM markers. Each column is one sample, and each cell represents a log2-transformed RMA-normalized expression value. Mean±s.d. (D-F,H) Unpaired, two-tailed *t*-test. ns, not significant; *P≤0.05; **P≤0.01.

Despite fibroblasts playing a key role in lung repair and fibrosis (Talbott et al., 2022; Wei et al., 2021; Wilson and Wynn, 2009), their functional role in asthma is relatively understudied. The ablated mice displayed no overt phenotype and lived a normal lifespan, as fibroblast ablation occurred after lung alveolization was completed. These mice are therefore suitable for establishing an asthma model to investigate the role of PDGFRα^+^ fibroblasts in ECM remodeling and disease progression. We used continuous house dust mite (HDM) exposure three times per week for 5 weeks to establish chronic asthma (Fig. 4A). Unsurprisingly, alveolar fibroblasts (tdTomato^+^ cells) did not expand numbers in response to chronic HDM exposure, regardless of PDGFRα^+^ fibroblast ablation (Fig. 4B-D). These data suggest that alveolar fibroblasts do not significantly contribute to tissue repair under chronic HDM exposure, with the established finding that airway remodeling is a major driver of asthma pathogenesis.

Chronic HDM exposure induced mild lung fibrosis in both control and ablated mice (Fig. 4E). We next determined elastin expression by qPCR, and ablated HDM-exposed lungs showed increased elastin expression (Fig. 4F). However, elastin staining of the lung tissues and quantification of elastin in lung lysates appeared similar between the control and ablated lungs exposed to chronic HDM, although there was a trend toward increased elastin levels in HDM-exposed ablated lungs (Fig. 4G,H). To gain further insights into alterations in ECM gene expression after HDM exposure, we conducted microarray analysis to identify DEGs for fibroblast/ECM markers. In Fig. 4I, ablated lungs had reduced expression of fibroblast or fibroblast/ECM-related genes, including *Tcf21*, *Pdgfra*, *Wnt2*, *Spon1*, *Mfsp4* and *Lox*. However, expression of a few ECM-related genes, such as *Fgg* and *Lum*, was higher in the ablated lungs. Taken together, these results suggest that fibroblast reduction leads to alterations in ECM production/composition in the lungs.

### PDGFRα^+^ fibroblast ablation promotes pathological features of asthma

**Fig. 5.**
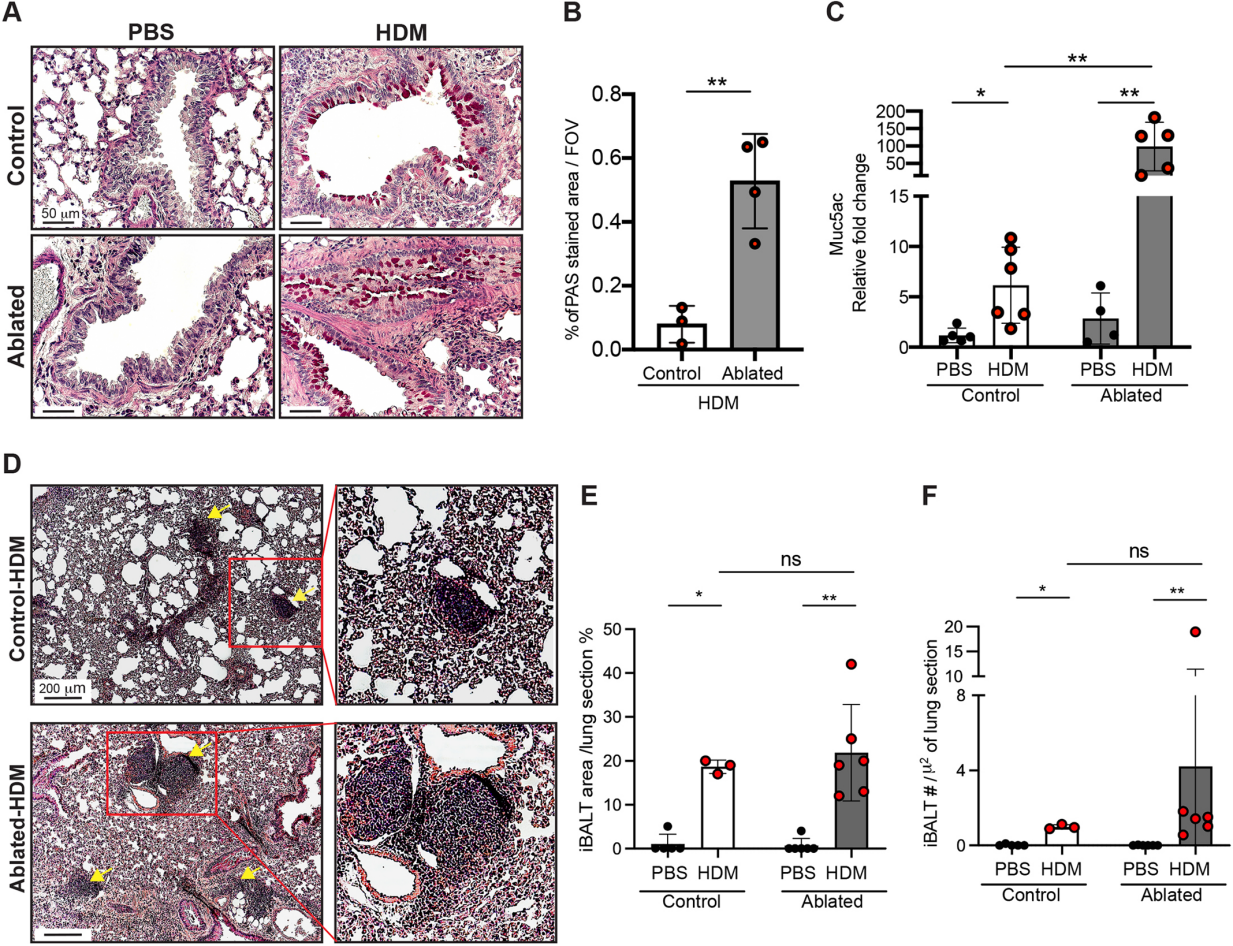
**Asthma pathology after chronic HDM exposure.** (A) Representative images of periodic acid–Schiff (PAS)-stained lung sections from PBS- or HDM-exposed control and ablated mice (*n*=5-6 per group). Scale bars: 50 µm. (B) Quantification of PAS-stained area in the lung sections. (C) qPCR analyses of *Muc5ac* normalized to *Gapdh*. Fold change compared to PBS-exposed control. (D-F) Inducible bronchus-associated lymphoid tissue (iBALT) formation in the lung. (D) Representative images of H&E-stained lung sections from HDM-exposed control and ablated mice. Yellow arrows indicate the iBALT structure. The right column shows magnified images of the boxed areas in the left column, containing iBLAT. Scale bars: 200 µm. (E,F) Quantification of iBALT area (E) and numbers (F) in PBS- and HDM-exposed control and ablated lung sections. Mean±s.d. (B,C,E,F). Unpaired, two-tailed *t*-test. ns, not significant; *P≤0.05; **P≤0.01.

Histological analysis of the HDM-exposed lungs demonstrated increased periodic acid–Schiff (PAS) staining in the airway epithelium in both control and ablated lungs, suggesting airway mucus hypersecretion (Fig. 5A). Notably, PAS^+^ areas were significantly increased in HDM-exposed ablated compared to HDM-exposed control lungs (Fig. 5B). Consistent with the increased PAS staining, we found that *Muc5ac* mRNA levels were elevated in the HDM-exposed lungs, compared to those in PBS-exposed lungs. Moreover, the HDM-exposed ablated lungs exhibited 10-fold greater *Muc5ac* expression than control HDM-exposed lungs (Fig. 5C). Inducible bronchus-associated lymphoid tissue (iBALT) formation can occur in response to various pathological processes, including inflammation, infection or chronic allergen exposure (Foo and Phipps, 2010; Silva-Sanchez and Randall, 2020; Eddens et al., 2017; Elliot et al., 2004). We observed distinct iBALT in the perivascular, peribronchial and bronchial areas in HDM-exposed control and ablated lungs. Interestingly, iBALT structures in the HDM-exposed ablated mice were slightly more plentiful and larger than those in controls (Fig. 5D-F). Taken together, our results demonstrate that fibroblast ablation before the onset of allergenic asthma leads to increased mucus secretion and *de novo* lymphoid tissue formation.

### PDGFRα^+^ fibroblast ablation promotes airway inflammation in HDM–exposed mice

**Fig. 6.**
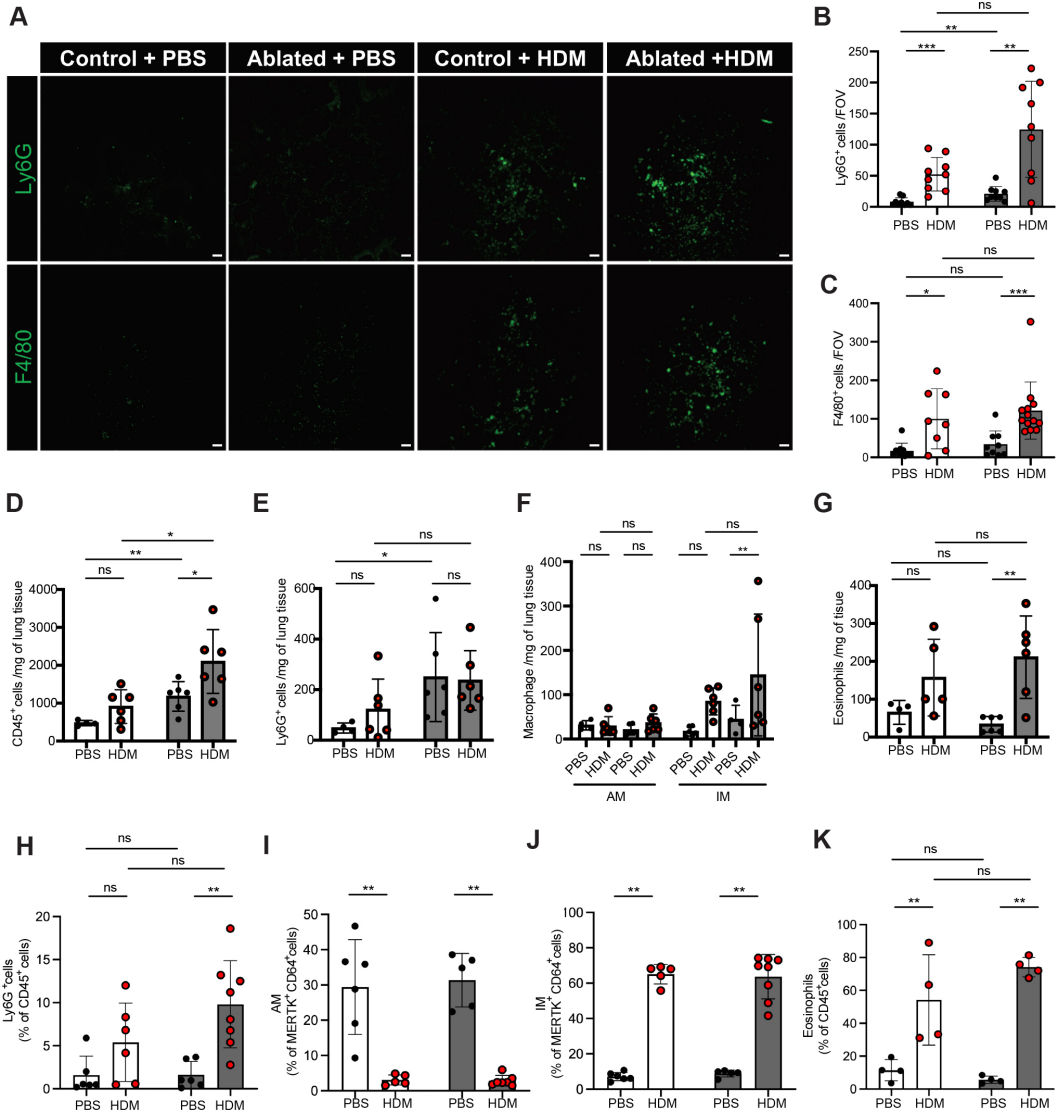
**Alterations in innate immune cells after chronic HDM exposure.** (A) Representative immunofluorescent staining of Ly6G (neutrophils) and F4/80 (macrophages) (*n*=4-6). Scale bars: 100 µm. (B,C) Quantification of Ly6G^+^ (B) and F4/80^+^ (C) area in the lung sections. (D-G) Flow cytometry analysis of CD45^+^ cells (D), Ly6G^+^ cells (E), alveolar macrophages (AM; MERTK^+^/CD64^+^/CD11b^−/low^CD11c^+^ cells) and interstitial macrophages (IM; MERTK^+^/CD64^+^/CD11b^high^CD11c^low^) (F), and eosinophils (G). (H-K) Flow cytometry analysis of BALF for the indicated cell types. Percentages of Ly6G^+^ cells (H), alveolar macrophages (I), interstitial macrophages (J), and eosinophils (K) are shown. Mean±s.d. (B-K) Unpaired, two-tailed *t*-test. ns, not significant; *P≤0.05; **P≤0.01; ***P≤0.001.

Fibroblasts can interact with immune cells, and this crosstalk influences functionality in a bidirectional fashion (Smolgovsky et al., 2024). Given elevated neutrophil counts in the ablated lungs at baseline (Fig. 3A,B), we next evaluated the effects of PDGFRα^+^ fibroblast ablation on innate immune response following chronic HDM exposure. We observed an increase in Ly6G^+^ cells (neutrophils) and F4/80^+^ cells (macrophages) in HDM-exposed ablated lungs compared to HDM-exposed control lungs (Fig. 6A-C). Flow cytometry confirmed that the ablated lungs exhibited an altered immune profile, both at baseline and in response to HDM exposure. Consistent with baseline lung flow analysis, HDM exposure in ablated mice resulted in an increase in leukocytes and neutrophils (Fig. 6D,E). Furthermore, interstitial macrophages (MERTK^+^/CD64^+^/CD11b^high^CD11c^low^) and eosinophils [CD11b^+^/Siglec-F^+^ (also known as Siglec5^+^)/Ly6G^−^] were significantly increased in HDM-exposed ablated lungs compared to PBS-exposed ablated lungs. A similar trend was observed in HDM-exposed control lungs, although the increase was not statistically significant (Fig. 6F,G). Similarly, analysis of BALF cells showed that the percentages of interstitial macrophages and eosinophils were significantly increased, but the percentages of alveolar macrophages were decreased, in HDM-exposed lungs compared to PBS-exposed lungs. BALF neutrophils were increased in HDM-exposed mice compared with PBS-exposed mice, and this effect was more pronounced in the ablated mice (Fig. 6H-K). Luminex analysis of BALF cytokines showed that IFN-γ, IL-1β and RANTES levels were significantly increased, and MCP-1 showed a trend toward an increase, in PBS-exposed ablated mice compared with PBS-exposed control mice (Fig. S3A,B,E,F). In HDM-exposed lungs, IL-1β, IL-2 and IL-10 levels were elevated compared with those in PBS-exposed lungs. However, the increase in IL-2 did not reach statistical significance owing to higher baseline levels in PBS-exposed ablated mice (Fig. S3B-D). Hierarchical clustering of all analytes across groups showed overall higher levels of inflammatory cytokines in PBS-exposed ablated mice compared with those in PBS-exposed control mice, which were further heightened by HDM exposure in ablated mice (Fig. S3G). These data suggest that PDGFRα^+^ fibroblast ablation under chronic allergen exposure increases airway inflammation.

To better understand the biological effects of PDGFRα^+^ fibroblast ablation in the HDM-induced asthma model, we performed microarray analysis to characterize the transcriptomic alterations in chronic asthma, which can provide insight into identifying genes/pathways related to inflammation. A total of 53 genes were significantly differentially expressed, with nine genes being upregulated and 44 genes being downregulated (|log_2_FC|≥1, adjusted *P*-value<0.05) in the HDM-exposed ablated lungs compared to HDM-exposed control lungs. Although there was no difference in *Muc5ac* and intelectin (*Itln1*) expression, the expression of *Gp2*, a marker of microfold (M) cells, was significantly increased by ∼3-fold in the HDM-exposed ablated lungs compared to HDM-exposed control lungs (Fig. S4A,B). M cells, a specialized epithelial cell commonly associated with mucosa-associated lymphoid tissues and other tertiary lymphoid structures, are responsible for antigen uptake and presentation to initiate immune responses against mucosal pathogens (Dillon and Lo, 2019; Kim and Jang, 2014), suggesting possible involvement of these cells in the innate and adaptive immune responses to chronic HDM exposure.

**Fig. 7.**
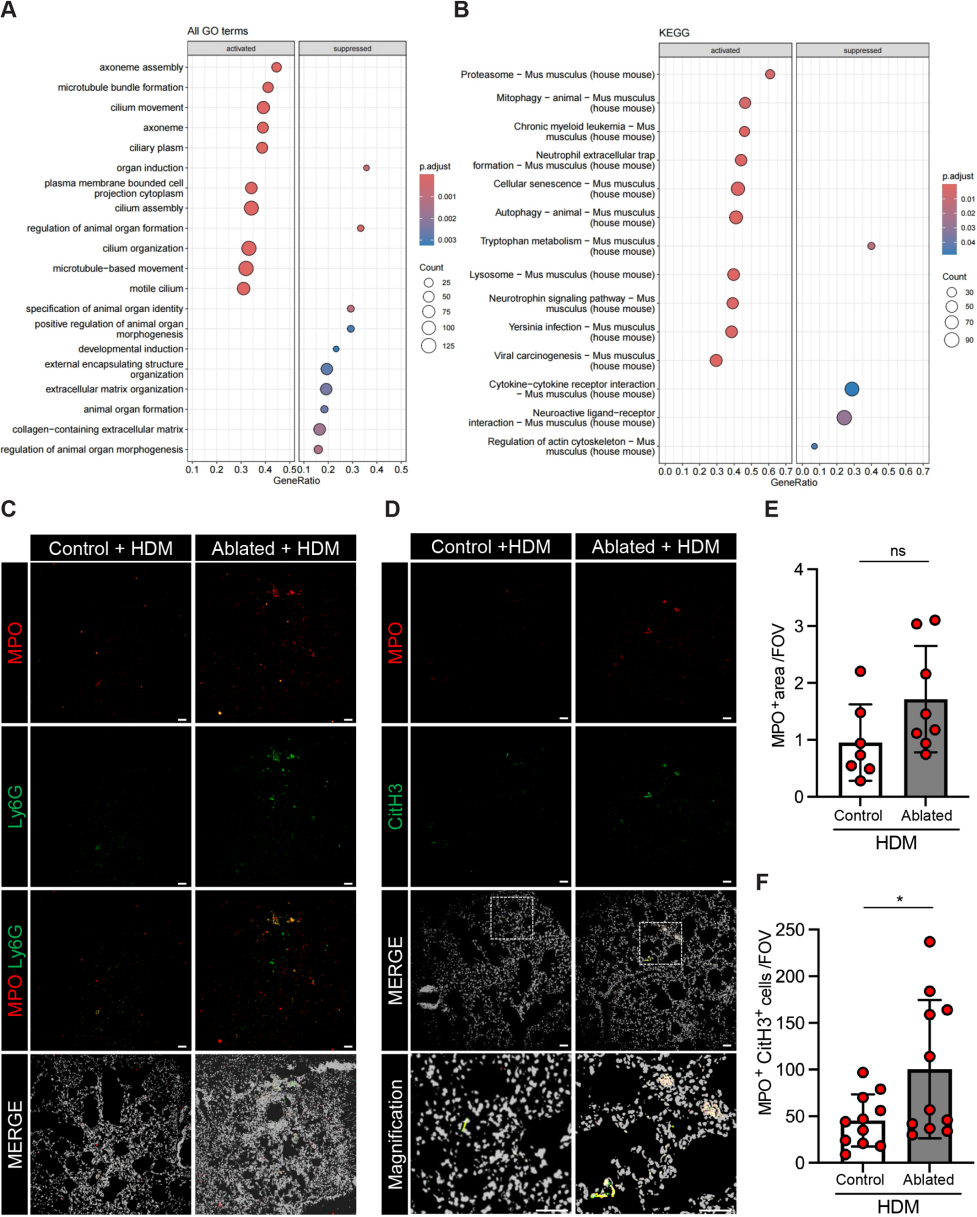
**Neutrophil activation following chronic HDM exposure.** (A,B) Dot plots displaying the results from gene set enrichment analysis performed on all genes sorted by log_2_FC. Significantly enriched Gene Ontology (GO) terms (A) and significantly enriched Kyoto Encyclopedia of Genes and Genomes (KEGG) pathways (B) in the PDGFRα^+^ fibroblast-ablated group (adjusted *P*-value<0.05) are shown. (C) Representative immunofluorescent staining of MPO^+^ cells (red), Ly6G^+^ cells (green) and merged images with DAPI (pseudocolor). Scale bars: 100 µm. (D) Representative immunofluorescent staining of MPO^+^ cells (red), CitH3^+^ cells (green) and merged images with DAPI (pseudocolor). Boxed areas are shown magnified below. Scale bars: 100 µm. (E) Quantification of MPO^+^ area. (F) Quantification of MPO^+^ and CitH3^+^ cells in the lung sections. *n*=3-5 mice per group, at least two fields per mouse, and each data point is one field. Mean±s.d. (E,F) Unpaired, two-tailed *t*-test. ns, non-significant; *P≤0.05.

To determine whether inflammatory pathways are enriched in the HDM-exposed ablated lungs, we performed gene set enrichment analysis (GSEA) to identify significantly enriched gene sets involved in inflammation. We observed significant enrichment of inflammation-related pathways, particularly leukocyte activation involved in inflammatory response, which was significantly upregulated in the HDM-exposed ablated lungs (Fig. S4C). Also, genes associated with the regulation of the inflammatory response pathway and the negative regulation of the inflammatory response were enriched in the HDM-exposed ablated lungs compared to the HDM-exposed control lungs (Fig. S4D,E). Gene Ontology (GO) analysis showed significant activation of pathways related to cilium function, including cilium movement, cilium assembly, cilium organization and motile cilium (Fig. 7A). Further analysis of ciliated epithelial markers (ciliated, goblet and club cells), annotated by single-cell transcriptomic profiling of the mouse lower respiratory tract (Martins et al., 2023), showed increased expression levels (Fig. S5). Interestingly, one of the enriched pathways identified by Kyoto Encyclopedia of Genes and Genomes (KEGG) was neutrophil extracellular traps (NETs), reflecting neutrophil activation, and studies have shown that excessive NET formation in the lungs has been reported in severe lung diseases, including asthma and chronic obstructive disease (Chacon et al., 2024; Grabcanovic-Musija et al., 2015). These data prompted us to further assess neutrophil activation and NET formation in HDM-exposed lungs (Fig. 7A,B). We found a significantly higher presence of activated neutrophils, identified as Ly6G^+^MPO^+^ cells, in the HDM-exposed ablated lungs compared to HDM-exposed control lungs (Fig. 7C). Further quantification of MPO^+^ area and NET-forming cells (MPO^+^CitH3^+^ cells) showed elevated NET levels in HDM-exposed ablated lungs compared to HDM-exposed control lungs (Fig. 7E,F). These data suggest that PDGFRα^+^ fibroblast loss promotes proinflammatory responses, at least partially through neutrophil activation and NET formation.

## DISCUSSION

Beyond fibroblasts' traditional roles in tissue repair and remodeling, research has demonstrated that they are crucial players in various biological processes, including immune regulation during inflammation. Owing to their diverse roles in diseases, targeting fibroblasts is likely to have both detrimental and beneficial actions for disease progression (e.g. adverse remodeling in fibrosis or accelerated growth in cancer) (Plikus et al., 2021; Talbott et al., 2022; Wei et al., 2021). Although our understanding of the contributions of fibroblasts to pulmonary fibrosis has advanced (Ghonim et al., 2023), fibroblasts’ role in asthma development remains relatively understudied. Thus, a better understanding of fibroblasts’ role in asthma is needed to determine whether targeting this cell type is beneficial in preventing disease progression. In this study, we dissected lung fibroblasts’ specific actions in allergic asthma development by establishing and studying mice with the loss of PDGFRα^+^ fibroblasts, known as resident lung fibroblasts. With this mouse model, our study showed that PDGFRα^+^ fibroblasts are not only responsible for ECM synthesis, homeostasis and remodeling in the lung, but also play a role in regulating immune response. In the homeostatic lung, the lung environment in the ablated mice is altered to favor immune cell recruitment. Furthermore, our data provide evidence that PDGFRα^+^ fibroblast loss favors the creation of neutrophilic conditions, accelerating asthma pathology through excessive mucus production, facilitated ECM alteration and increased NETs within the lung. This work extends knowledge of the role of PDGFRα^+^ fibroblasts in allergic airway disease, and loss of fibroblasts before the onset of asthma results in detrimental disease outcomes.

As expected, the histological structure of the ablated lung, compared to that of control lung, showed a thinner mesenchyme and a tendency toward larger alveolar spaces. Consistent with the previous finding of Col1a1-GFP^+^ cells co-expressing PDGFRα (Hung et al., 2013), ablation of PDGFRα fibroblasts drastically reduced *Col1a1* promoter activity throughout the mesenchyme, indicating that they are one of the fibroblast populations regulating *Col1a1* transcription. However, the overall collagen content in the baseline ablated lungs remained relatively well preserved. Studies showed that collagen turnover in the adult lung is relatively low (Burgess and Harmsen, 2022; Onursal et al., 2021). Our data suggest that type I collagen is relatively stable or that other cell types may contribute to ECM maintenance. These results are similar to those observed from studies in the heart (Kuwabara et al., 2022).

Another feature of ablated mice was deficiency of lipofibroblasts, a consequence of ∼50% of PDGFRα^+^ lineage cells differentiating into lipofibroblasts (Park et al., 2019). During bleomycin-induced lung fibrosis, lipofibroblasts can transdifferentiate into activated myofibroblasts depending on PPARγ signaling (El Agha et al., 2017) and then revert to a lipofibroblast-like phenotype during the resolution phase. These findings suggest that the loss of PDGFRα-lineage lipofibroblasts results in fewer myofibroblasts available to participate in tissue repair following HDM exposure. Impaired tissue repair leads to delayed resolution of inflammation and maladaptive remodeling, thereby contributing to asthma pathology.

Ablated mice showed increased baseline *Lum* expression in the lung. Furthermore, *Lum* was among the most upregulated DEGs in HDM-exposed lungs compared with HDM-exposed control lungs. Elevated *Lum* expression, in turn, can promote fibroblast activation, fibrocyte differentiation and epithelial–mesenchymal transition (Li et al., 2013; Pilling et al., 2015; Wang et al., 2022). Therefore, the altered expression of ECM genes observed in ablated lungs may impair ECM remodeling and tissue repair, but also induce fibroblast activation during chronic HDM exposure, thereby influencing airway function. Several studies have shown airway dysfunction in mice chronically exposed to HDM, with methacholine-challenged mice exhibiting bronchoconstriction and increased airway resistance consistent with the pathological airway hyper-responsiveness characteristic of asthma progression (Saglani et al., 2009; Woo et al., 2018). Although worsened pathological features were observed in HDM-exposed ablated mice, further studies are needed to determine whether PDGFRα^+^ fibroblast loss alters lung physiology at baseline and during HDM exposure, in order to better understand the mechanisms underlying disease severity.

Accumulating evidence demonstrates that fibroblasts interact dynamically with immune cells, influencing lung immunity and the disease pathology (Hung et al., 2013; Smolgovsky et al., 2024; Wei et al., 2021). In the homeostatic state, neutrophil numbers were increased in the lungs and BALF in the ablated lungs, while macrophages and eosinophils remained comparable to those in the control lungs. In addition, when comparing BALF cytokines at baseline, overall inflammatory cytokine levels were higher in ablated mice than in controls. Transcriptomic analysis further highlighted dysregulated pathways related to inflammatory responses and leukocyte activation in ablated lungs at baseline. This suggests that PDGFRα^+^ fibroblast loss, even in the absence of lung injury, alters the lung environment to facilitate neutrophil recruitment but is less likely to recruit blood monocytes and eosinophils. Studies have shown that fibroblasts can directly and indirectly regulate immune responses by secreting ECM and ECM-bound molecules. ECM components provide an intrinsic signal to interact with immune cells and influence their phenotypes, either promoting or inhibiting the inflammatory responses (Sutherland et al., 2023; Wight et al., 2017). Thus, altered ECM components in the ablated lungs are likely part of regulating the perpetuation of immune cell compositions and their recruitment from the circulation. However, it remains mechanistically unclear how PDGFRα^+^ fibroblast loss leads to modulation of ECM and regulation of cytokines and chemokine production in homeostatic lungs, resulting in this observed altered recruitment.

Following chronic HDM exposure, the ablated lung environment showed increased immune cells – including neutrophils, eosinophils and monocytes – which differentiate into interstitial macrophages. This immune cell increase may worsen asthma pathology, evidenced by excessive mucus secretion, increased iBALT formation and a trend toward greater BALF cytokine levels in HDM-exposed ablated lungs compared to HDM-exposed control lungs. In HDM-ablated mice, inflammatory (IL-1β) and regulatory/activation-associated (IL-2 and IL-10) cytokines were increased in BALF. These findings suggest that our HDM model does not fully recapitulate a type 2-driven asthma phenotype but instead reflects non-type 2 asthma, often characterized by inflammasome activation, interferon signaling and IL-17 pathways (Liu et al., 2024). We cannot rule out the possibility that the mice on a mixed C57BL/6J background exhibit a different response to HDM (Whitehead et al., 2003). The influence of crossing different strains could have affected the outcome of the allergic immune response. We did not directly compare HDM exposure between our mixed background mice and C57BL/6 mice, which are generally more prone to neutrophilic inflammation in ovalbumin-induced asthma models (Whitehead et al., 2003), which may underlie the weaker IL-4, IL-5 and IL-13 responses observed. Another possibility is that the increased IL-10 levels in BALF from HDM-exposed mice compared to BALF from PBS-exposed mice suggest activation of regulatory pathways that may further dampen T helper (Th)2 effector responses (Branchett et al., 2020). Together, these results suggest that the worsened asthma pathology observed in ablated mice more closely resembles a neutrophil-driven inflammatory response rather than classical type 2 immunity. Non-type 2 asthma is frequently observed in patients with severe and/or steroid-resistant disease (Liu et al., 2024). Thus, further studies are warranted to investigate the molecular mechanisms driving this pathological phenotype, including inflammasome activation, Th1/Th2 skewing, and the modulation of inflammatory and immune responses under neutrophilic conditions in the lung.

NETs play a key role in the pathology of neutrophilic lung diseases, including asthma, chronic obstructive pulmonary disease and cystic fibrosis (Keir and Chalmers, 2022). Several clinical observations in asthma patients found higher NET levels in blood and the upper airway that are positively correlated with the severity of asthma (Varricchi et al., 2022; Wright et al., 2016). We found that NETs were detected in the lung parenchyma rather than in the airways. Although we cannot exclude the presence of NETs in the trachea, as it was not examined in this study, this finding suggests that parenchymal NETs may promote tissue damage and inflammation. A recent study showed that exposure of mice to low-dose LPS can cause a local increase in CXCR4^high^ neutrophils, which are prone to forming NETs in mice. Furthermore, released NETs can promote activation of dendritic cells and further augment allergic response in the HDM-exposed asthma model (Radermecker et al., 2019). These findings support that increased NETs in the ablated lung can facilitate airway inflammation and enhance susceptibility to allergic asthma through similar mechanisms. However, it remains unclear whether the elevated NET levels in HDM-exposed ablated lungs result from impaired NET clearance and/or increased local NET formation capacity.

In conclusion, our study provides evidence that PDGFRα^+^ fibroblasts play a crucial role in regulating immune homeostasis. Furthermore, the loss of PDGFRα^+^ fibroblasts worsens asthma pathology by creating a lung environment that elevates neutrophil numbers, their activation and mucus production. These findings open up new research avenues into fibroblast–immune crosstalk in asthma pathogenesis and disease progression, highlighting the potential for therapeutic strategies to modulate fibroblast function to alleviate allergic response, ultimately improving asthma outcomes.

## MATERIALS AND METHODS

### Mice

All animal protocols and procedures were approved by the University of Hawai'i Institutional Animal Care and Use Committees and were conducted in accordance with the National Institutes of Health (NIH) guidelines for care and use of laboratory animals. The *Gt(ROSA)26Sort*^dT^ (#007914) and *Gt(ROSA)26Sor^DTA^* (#010527) mice were purchased from The Jackson Laboratory (JAX). The *Pdgfra^CreERT2/+^* mice (Chung et al., 2018; Ivey et al., 2019) were kindly provided by Dr Brigid Hogan at Duke University Medical Center (now available at JAX #032770). The *Col1a1-GFP* transgenic mice, *Tg(Col1a1-EGFP)*, were generated by Dr David Brenner (Lin et al., 2008). Both male and female mice on a mixed C57Bl/6 background were used for the study, and no significant differences were observed for the parameters that were measured. All age-matched control mice, *Pdgfra^CreERT2/+^; Gt(ROSA)26Sor^tdT/+^* or *Pdgfra^+/+^; Gt(ROSA)26Sor^tdT/DTA^*, were tamoxifen induced. All experiments were performed on adult mice older than 8 weeks.

### Tamoxifen administration

Tamoxifen (MP Biomedicals, 0215673891; AdipoGen, 50-149-0595) was dissolved in sunflower seed oil containing 10% ethanol. Tamoxifen (0.2 mg/g body weight) was administered to adult mice (both male and female) aged between 8 and 10 weeks by oral gavage twice on nonconsecutive days. Control genotypes including *Pdgfra^CreERT2/+^; Gt(ROSA)26Sor^tdT/+^*, *Pdgfra^+/+^* and uninduced *Gt(ROSA)26Sor^tdT/DTA^* littermates were used. Following tamoxifen induction, mice were housed under standard conditions and used for either assessing fibroblast ablation efficiency or establishment of asthma model 3-7 months post-induction.

### HDM-induced allergenic asthma model

The HDM-induced allergenic asthma model was established as previously described (Malaviya et al., 2021) with the following modifications. 12.5 µl of PBS (vehicle) or HDM extract (Greer Laboratories Inc; 25 μg) was administered intranasally to anesthetized mice every other day for 5 weeks. Mice were killed within 48 h after the final treatment, and lung tissues and BALF were collected for further analysis.

### Reverse transcription qPCR

The superior lobes were stored in RNAlater (Invitrogen, AM7021) at −20°C, then homogenized in TRIzol Reagent (Thermo Fisher Scientific, 15596026). Total RNA was isolated according to the manufacturer's instructions. RNA quality and concentration were determined by NanoDrop ND-1000 (Thermo Fisher Scientific). Reverse transcription synthesis of first-strand cDNA from total RNA was performed using M-MLV Reverse Transcriptase and buffer (Sigma-Aldrich, M1302-40KU) and random hexamers (Thermo Fisher Scientific, S0142). qPCR analysis was performed with PowerUp SYBR Green Master Mix (Thermo Fisher Scientific, A25742) on a QuantStudio 5 instrument (Thermo Fisher Scientific, A34322). Samples were run in triplicate and normalized to *Gapdh* expression. The 2^− –ΔΔCt^ method was used to determine relative gene expression levels. Primer sequences are available upon request.

### Histology and immunostaining

Lungs were lavaged with Dulbecco's phosphate-buffered saline (DPBS) via the trachea and then perfused through the right ventricle of the heart, and fixed with 1% or 4% paraformaldehyde (PFA) at 4°C overnight. Lung tissues were then processed for either frozen or paraffin embedding. Paraffin sections (5 μm) were permeabilized in 0.1% Triton X-100 (Sigma-Aldrich) for 10 min, then blocked for 1 h with blocking buffer containing 1% bovine serum albumin (BSA) and 1.5% goat or donkey serum. Sections were incubated with primary antibodies (anti-Plin2, ab52356, Abcam, 1:500 dilution; anti-Lum, AF2745, R&D Systems, 1:100 dilution) at 4°C overnight. Secondary antibodies coupled to Alexa Fluor 488 or 594 (1:500 dilution; Thermo Fisher Scientific) were used. Sections were counterstained with 4′,6-diamidino-2-phenylindole (DAPI; Roche, 10-236-276-001) for 15 min at room temperature (RT). For histological examination, H&E (ChromaView, 88028 and 88029), Gomori trichrome (Volusol, VXT-032), PAS (Polysciences, 24220) and Verhoeff Van Gieson elastin (Ethos Biosciences, 25089) staining were performed according to standard protocols.

### Hydroxyproline assay

Hydroxyproline assay (Cell Biolabs, STA-675) was performed on right lung lobes isolated from mice. Briefly, 15-20 mg tissue was homogenized in deionized water at a ratio of 10 mg tissue per 100 µl water. 125 µl of 12 N HCl was added to the same volume of homogenate and digested at 120°C for 4 h. Hydrolyzed contents were processed according to the manufacturer's instructions. Samples and standards were added to a 96-well microplate, and absorbance was measured at 540-570 nm using a SpectraMax M3 microplate reader (Molecular Devices). Hydroxyproline concentrations were normalized to protein concentration, which were determined using Pierce™ BCA Protein Assay Kits (Thermo Fisher Scientific, 23227)

### Western blotting

Whole-protein lysates from snap-frozen right lung tissues were prepared in RIPA buffer (25 mM Tris-HCl pH 7.6, 150 mM NaCl, 1% NP-40, 1% sodium deoxycholate, 0.1% SDS) with protease inhibitor cocktail (Bimake, B14001). Lysates were immunoblotted with the following antibodies: anti-Tcf21 (PA5-116012, Thermo Fisher Scientific, 1:200 dilution), anti-PDGFRα (AF1062, R&D Systems, 1:100 dilution) and anti-Plin2 (ab52356, Abcam, 1:500 dilution). The membranes were then incubated with the corresponding Li-Cor IRDye secondary antibodies for 1 h at RT. Membranes were imaged on a Li-Cor Odyssey CLx, and images were analyzed using Image Studio version 5.2.5 software (Li-Cor).

### Flow cytometry analysis of lung tissues and BALF cells

Lung single-cell suspensions were obtained as previously described (Park et al., 2019). Briefly, the right lobes were excised and immediately transferred to 2 ml digestion buffer: DPBS containing 0.9 mM Ca^2+^, collagenase type IV (600 U/ml; Worthington, LS004188) and dispase II (1.2 U/ml; Thermo Fisher Scientific, 17105041). Tissue was minced into ∼1-2 mm pieces and incubated at 37°C for 45 min with gentle agitation. Enzymatic activity was inhibited by adding 5 ml DPBS, and the cell suspensions were sequentially filtered through 40-μm and 30-μm filters and washed in 1% BSA-DPBS. All samples were processed using the same lobe, enzyme concentrations, volumes and incubation conditions.

BALF was collected from the whole lungs by infusing 1 ml PBS via a tracheal cannula, repeated three times, then the BALF was centrifuged at 460 ***g*** for 5 min. The supernatants were stored at −80°C for Luminex analysis. The cell pellets were treated with TheraPEAK^®^ ACK Lysing Buffer (Lonza Bioscience, BP10-548E) for 5 min at RT, followed by washing with PBS. A LIVE/DEAD™ Fixable Yellow Dead Cell Stain Kit (Thermo Fisher Scientific, L34959) was used for live/dead discrimination. Cells were then incubated in Fc block (1:100; Tonbo Bioscience, 70-0161 U500) followed by the indicated antibody cocktail and fixed with 4% PFA in flow cytometry buffer (1% BSA-DPBS) for 10 min at RT. Primary antibodies used for flow cytometry were as follows: panel 1: anti-CD45 Pacific blue (1:500; BioLegend, 157212), anti-MERTK APC (1:200; BioLegend, 151508), anti-CD64 PE cy7 (1:100; BioLegend, 139314), anti-CD11b-BV711 (1:500; BioLegend, 101241), anti-CD11c-PerC/Cyanine5.5 (1:400; BioLegend, 117327); panel 2: anti-CD45 Pacific blue (1:500; BioLegend, 157212), anti-CD11b-PerCP/Cyanine5.5 (1:500; BioLegend, 101228), anti-Ly6G BV711 (1:150; BioLegend, 127643), anti-CD170 APC. (1:200; BioLegend, 155507). AccuCheck counting beads (Thermo Fisher Scientific, PCB-100) were added according to the manufacturer's protocol prior to acquisition. Data were acquired on an LSRFortessa (BD Bioscience) and analyzed using FlowJo software version 10 (BD Bioscience). Cell counts were calculated according to the AccuCheck counting bead manufacturer's recommendations and normalized to the weight of each lung. Compensation beads (eBioscience, 01-2222-42) were used for antibody compensation analysis. Data were acquired on a LSRFortessa (BD Bioscience). Gating analyses were performed using FlowJo (TreeStar).

### Gene expression profiling and microarray data processing

The right upper lobes were used for microarray, and RNA extraction was performed as described above. Clariom S Assay mouse microarray (Thermo Fisher Scientific, 902930) was conducted at the Genomic and Bioinformatics Shared Resource at the Cancer Center at the University of Hawai’i. The analysis of the Affymetrix Clariom S mouse CEL files was conducted using R statistical software version 4.3.3. The raw dataset, provided in CEL-file format, was imported and analyzed using the read.celfiles function of the Bioconductor oligo package (version 1.66.0). These data were pre-processed using the robust multi-array averaging (RMA) method provided by oligo, and the adjusted expression table was formatted as probe ID by samples. Principal component analysis was performed on the normalized expression values of PDGFRα^+^ fibroblast-ablated and control samples using the prcomp function in R. The resulting data were plotted using ggplot2 (v3.5.0).

Empirical Bayes smoothing for transcripts with multiple probes was performed using the Linear Model for Microarray Analysis (LIMMA) package (v.3.58.1). LIMMA employs empirical Bayes techniques for differential expression analysis, allowing the identification of gene expression differences between PDGFRα^+^ fibroblast-ablated lung and control lung samples (Ritchie et al., 2015). Genes with significant differences, defined as an absolute log_2_FC greater than 1 and an adjusted *P*-value less than 0.05, were considered DEGs. For visualization of airway epithelial cell marker gene expression, genes with |log2FC| greater than 0.5 were selected from the differential expression results comparing HDM-exposed control and ablated mice. Airway epithelial cell markers – including those associated with ciliated, goblet and club cells – were curated based on the single-cell transcriptomic profiling of the mouse lower respiratory tract reported by Martins et al. (2023). Heatmaps were generated using the pheatmap package (v1.0.12). A heatmap of genes related to fibroblasts and the ECM was generated using the pheatmap package (v.1.0.12). The expression levels of all probes were normalized, and analysis was conducted using LIMMA.

For pathway analysis between PDGFRα^+^ fibroblast-ablated and control groups under PBS and HDM conditions, DEGs were ranked according to their log_2_FC values. GSEA was performed using ClusterProfiler (v.4.10.1) (Wu et al., 2021; Yu et al., 2012). The gseGO and gseKEGG functions of ClusterProfiler were employed to analyze GO terms within the Biological Process (BP), Molecular Function (MF) and Cellular Component (CC) categories, as well as KEGG pathways (Kanehisa et al., 2016). The org.Mm.eg.db (v.3.18.0) database was utilized for pathway and gene information. *P*-values were adjusted using the Benjamini–Hochberg method, and results with adjusted *P*-values below 0.05 were considered significant.

### MLI measurement

The MLI was measured according to a previously reported method for semi-automated quantification of MLI (Crowley et al., 2019). Briefly, randomly selected five-field H&E- or trichrome-stained images from the left lung of each mouse were analyzed with Fiji/ImageJ version 1.54p. Briefly, the images were converted to 8-bit images, then Huang thresholding binarized the image to denote airspace and lung. Each binarized image was overlaid with eight semi-transparent horizontal and vertical chords on binarized images. Discrete chords traversing alveolar septa, isolated based on pixel, were measured, and the mean of these chord lengths was exported from ImageJ into Microsoft Excel sheets. Data were analyzed with GraphPad Prism 10.6.0.

### Whole lung clearing

Lungs were inflated with DPBS, fixed in 4% PFA overnight at 4°C, then cleared with Ce3D™ tissue clearing solution (BioLegend, 427702) on a rocker protected from light for 3 weeks.

### Quantification of protein levels in BALF and lung lysates

Lung tissue lysates were prepared in cell lysis buffer (Cell Signaling Technology, 9803) with 1 mM phenylmethylsulfonyl fluoride (Fisher Scientific, 50-136-8487). Proteins were used to measure mouse elastin levels by enzyme-linked immunosorbent assay (Novus Biologicals, NBP3-06918). Cytokine levels in BALF were measured using remixed 25 Plex-Immunology Multiplex Assay Mouse Cytokine/Chemokine Magnetic Bead Panel (MilliporeSigma, MCYTOMAG-70K-PMX) according to the manufacturer's instructions.

### Imaging and statistical analysis

A Zeiss Axiovert 200M was used for fluorescence imaging, and a Zeiss Axioskop 2 Plus was used for bright-field images. Whole-lung fluorescent images were obtained from the Leica Thunder 3D Live Cell Imaging System through maximum-intensity projections taken at 10×. In the majority of figures, the left lung was imaged. For tdTomato^+^ cells quantification, four fields per section from three nonconsecutive sections were imaged. Images were normalized as stated in the figure legends.

For iBALT quantification, histology slides of the left lung lobe were scanned using an AxioScan 7 slide scanner (Zeiss) and analyzed with ZEN Lite software (version 1.3.25057.2). iBALTs greater than 120 µm in diameter were identified by dense nuclear staining of lymphoid aggregates colocalized to bronchioles. The Contour (Spline) function was used to outline the iBALT and lung section and the area. Percentages were calculated by dividing iBALT area by the lung section area. ImageJ software was used for the remaining image quantification. All statistical calculations were performed using Prism 9 (GraphPad). All data are represented as mean±s.d., and biological replicates are shown. For analyses conducted using GraphPad Prism, unpaired, two-tailed Student's *t-*test was used to compare two groups, and one-way ANOVA with post hoc Tukey's test or two-way ANOVA with post hoc Šidák's test was used to compare multiple groups. *P*-value notation in figure legends is as follows: not significant (n.s.) *P*>0.05; *P≤0.05; **P≤0.01; ***P≤0.001.

## Supplementary Material

10.1242/dmm.052323_sup1 Supplementary information
