# Supplementary material for: PDGFRα+ fibroblast ablation exacerbates pathologic features in a model of house dust mite-induced allergenic asthma
Source: Dis Model Mech. 2025 Dec 29;18(12):dmm052323. doi: 10.1242/dmm.052323 (PMC12805647; doi:10.1242/dmm.052323)
Supplement: Supplementary information [file dmm-18-052323-s1.pdf]

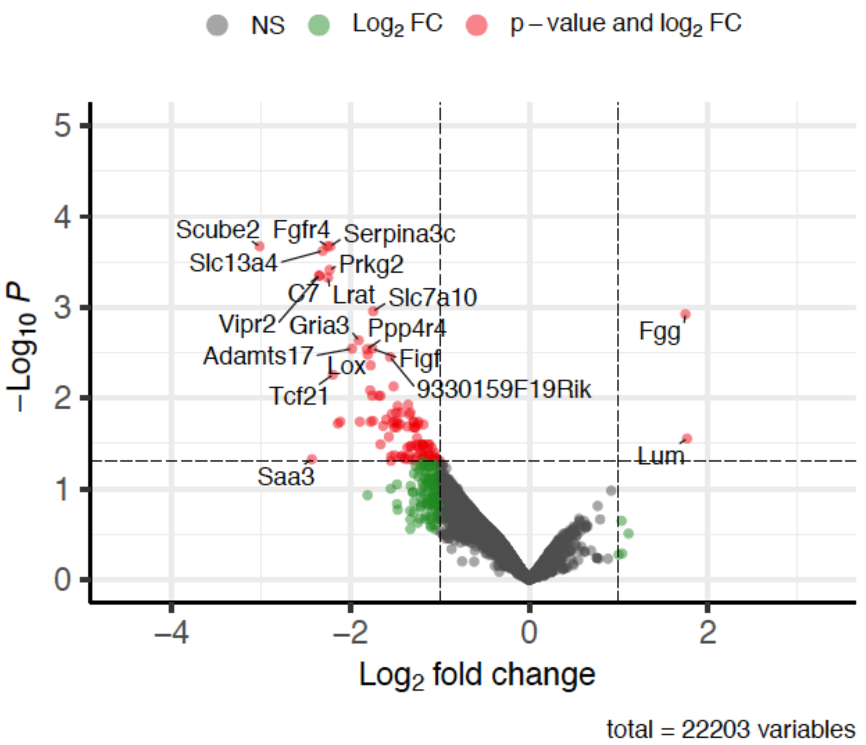

Fig. S1. Volcano plot of DEG. Genes with |log2FC| > 1 and adjusted p-value > 0.05 are highlighted in green, while genes that satisfy both |log2FC| > 1 and adjusted p-value < 0.05 (DEGs) are highlighted in red.

Disease Models & Mechanisms • Supplementary information

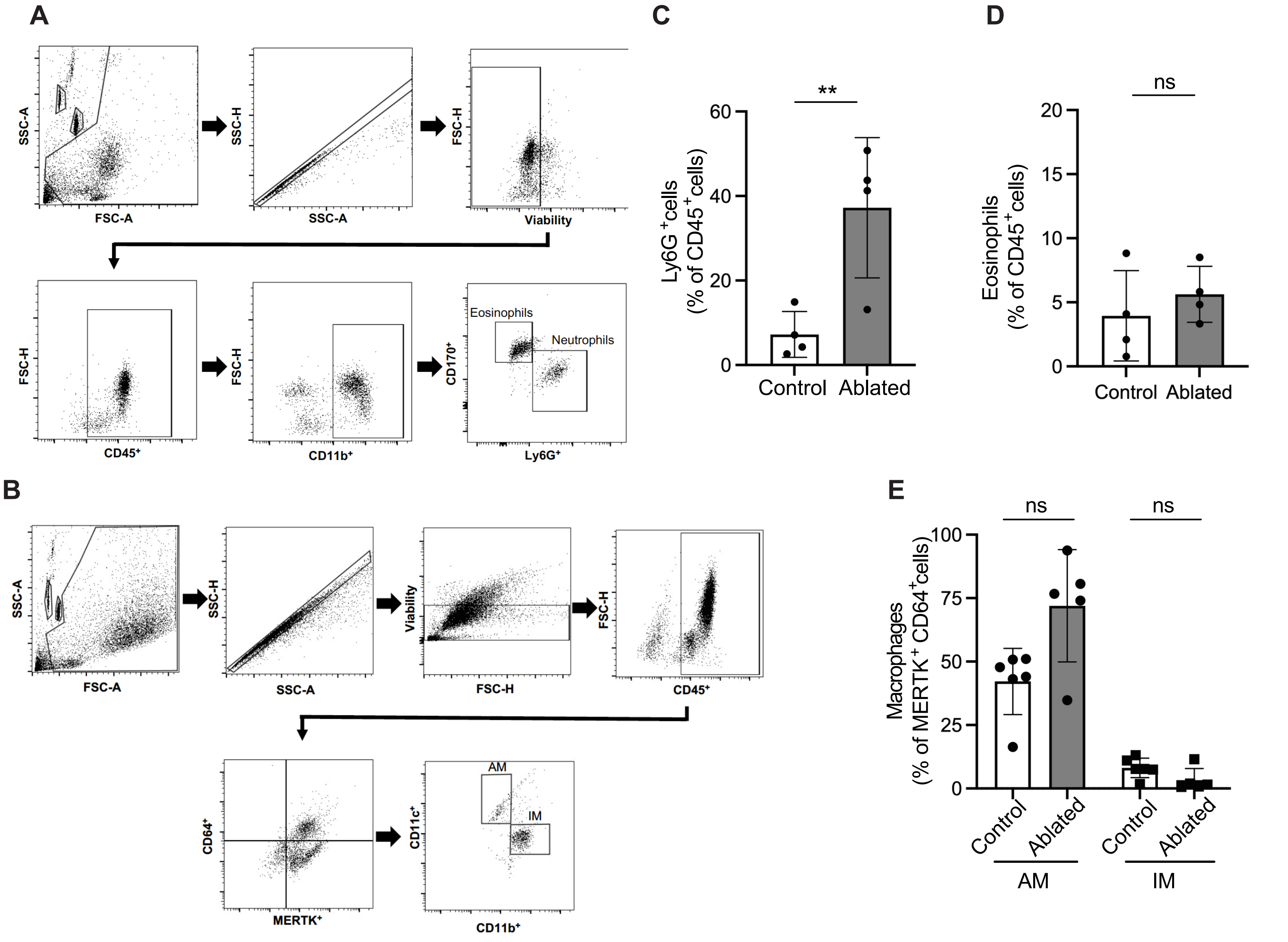

Fig. S2. A-B: Gating strategy for identifying eosinophils ($CD45^+CD11b^+Ly6G^-CD170^+$), neutrophils ($CD45^+CD11b^+Ly6G^+CD170^-$) (A), and alveolar macrophages (AM; $MERTK^+CD64^+CD11^{blow/-}CD11c^+$) and interstitial macrophages (IM; $MERTK^+CD64^+CD11b^{high}CD11c^{low}$) (B). C-E: Immune cell composition of BALF at baseline.(C) Percentage of Ly6G+ neutrophils among $CD45^+$ cells. (D) Percentage of eosinophils among $CD45^+$ cells. (E) Percentages of AM and IM among $MERTK^+CD64^+$ cells. Data are shown as mean ± SD. Unpaired t-test; ns, not significant; $^{**}p \leq 0.01$

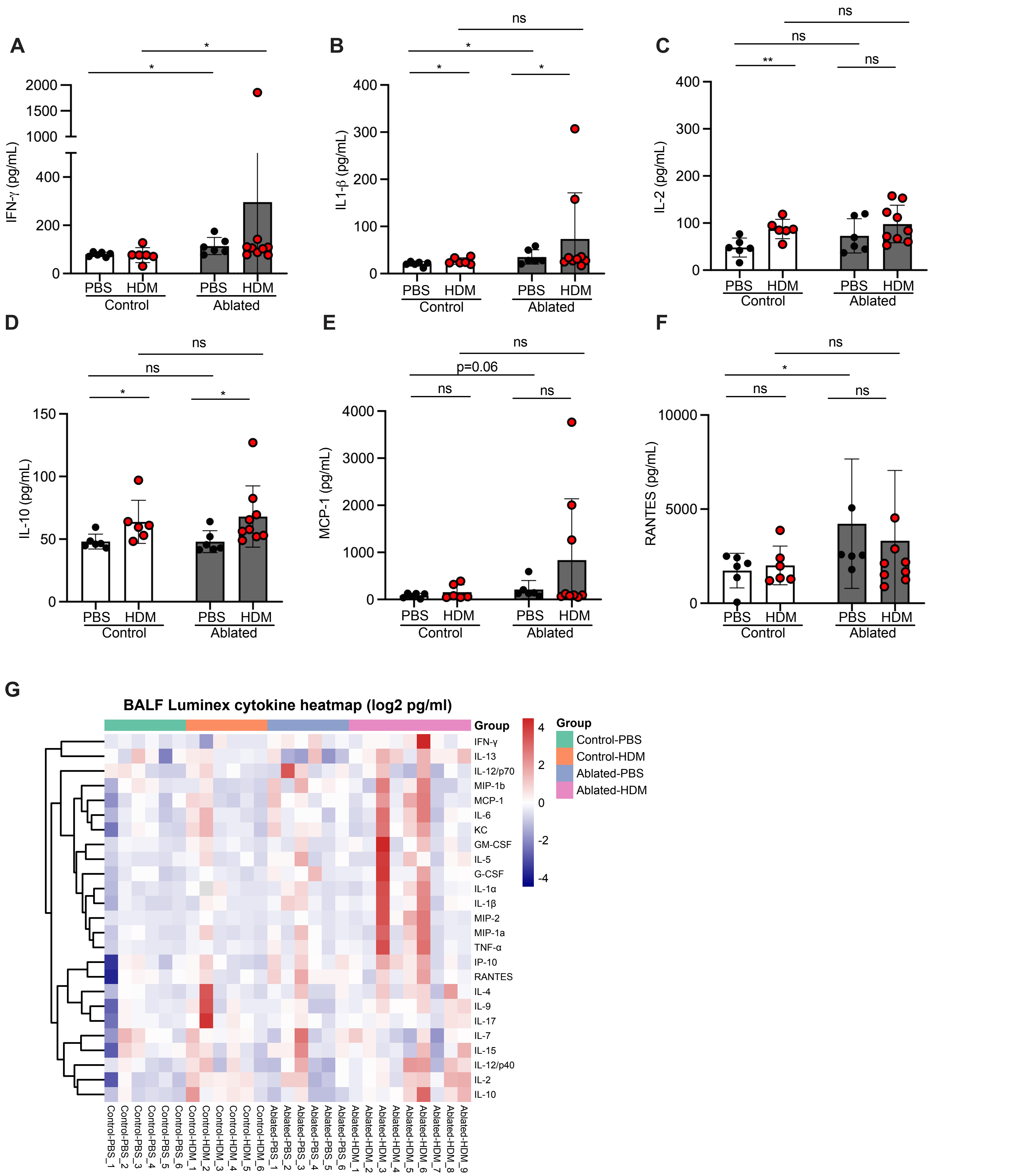

Fig. S3. A-G: BALF cytokine levels in PBS- or HDM-exposed control and ablated mice. (A) IFN-γ, (B) IL-1β, (C) IL-2, (D) IL-10, (E) MCP-1, (F) RANTES, and (G) heatmap of BALF cytokines (log2 pg/mL). Data are shown as mean ± SD. Unpaired t-test; ns, not significant; * $p \leq 0.05$ ** $p \leq 0.01$.

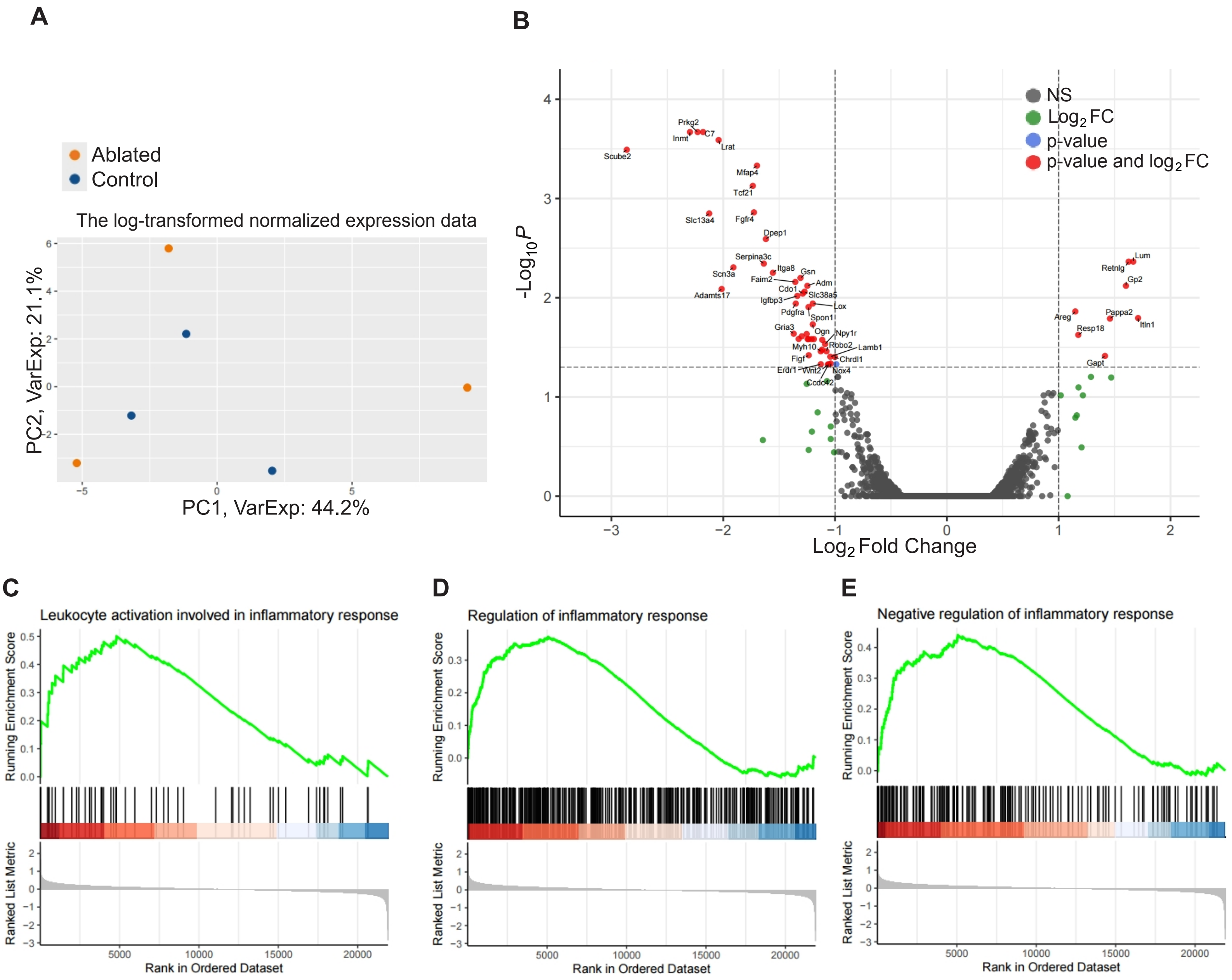

Fig. S4. Microarray analysis in the baseline control and ablated lungs exposed to HDM.

A: PCA plot of the normalized expression values of ablated and control samples.

B: A volcano plot visualizing DEGs between the ablated and control groups. Significant genes are highlighted as in Figure 2-C. C-E: GSEA plots displaying significantly activated proinflammatory pathways (C) Leukocyte activation involved in inflammatory response (D) Regulation of inflammatory response (E) Negative regulation of inflammatory response in the ablated group.

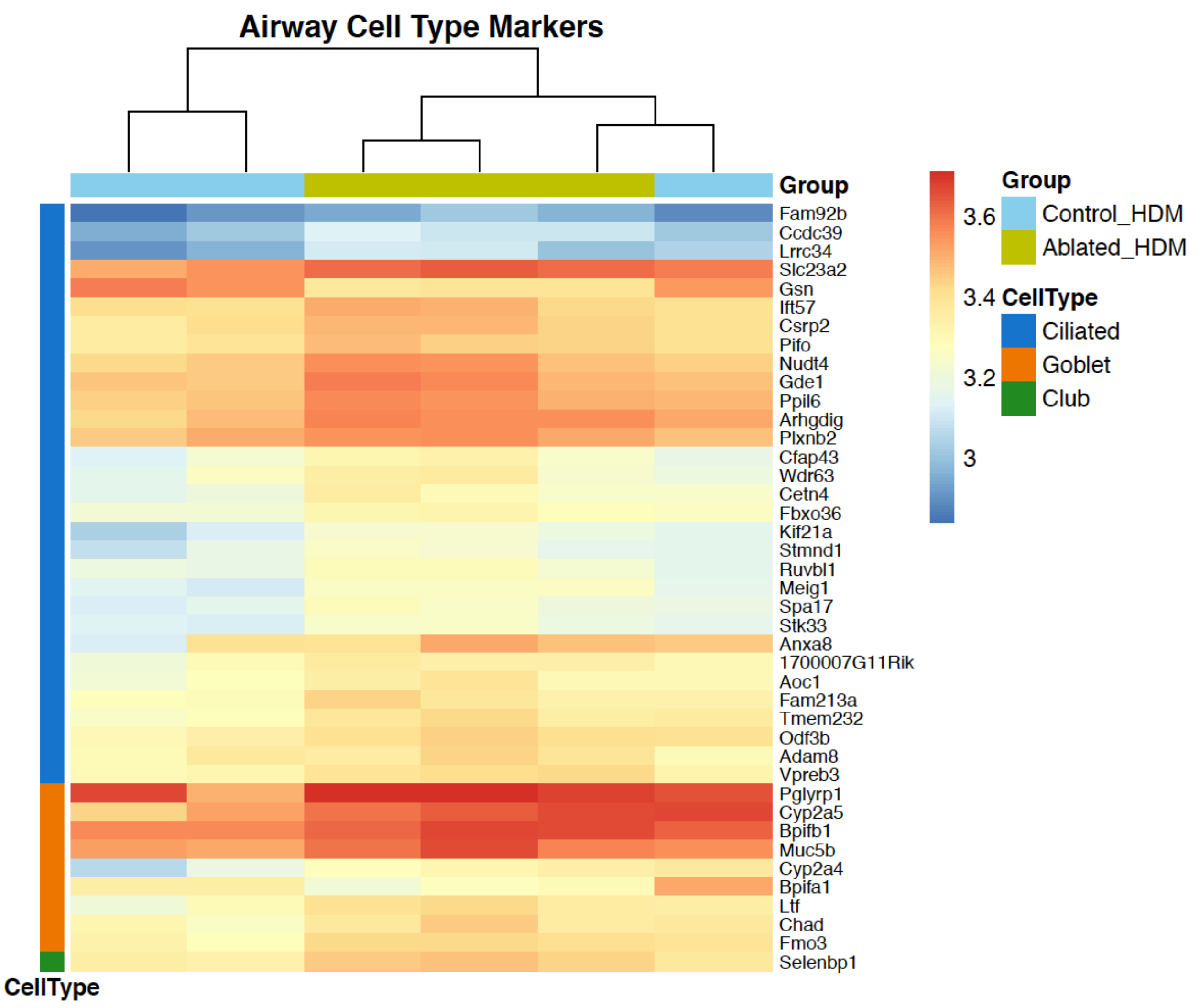

Fig. S5. Heatmap of airway epithelial cell type marker genes filtered by |log2 fold change| > 0.5 between HDM-exposed control and ablated mice.

Disease Models & Mechanisms • Supplementary information
